# EURO-WABB: an EU rare diseases registry for Wolfram syndrome, Alström syndrome and Bardet-Biedl syndrome

**DOI:** 10.1186/1471-2431-13-130

**Published:** 2013-08-27

**Authors:** Amy Farmer, Ségolène Aymé, Miguel Lopez de Heredia, Pietro Maffei, Susan McCafferty, Wojciech Młynarski, Virginia Nunes, Kay Parkinson, Véronique Paquis-Flucklinger, Julia Rohayem, Richard Sinnott, Vallo Tillmann, Lisbeth Tranebjærg, Timothy G Barrett

**Affiliations:** 1 Birmingham Children’s Hospital, Steelhouse Lane, Birmingham B4 6NH, UK; 2 INSERM - SC11, Platforme Maladies Rares, 96 Rue Didot, Paris 75014, France; 3 IDIBELL, Hospital Duran i Reynals, 3ª Planta, Gran Via de L’Hospitalet, 199, E-08907- L’Hospitalet de Llobregat, Barcelona, Spain; 4 Centro de Investigación en Red de Enfermedades Raras (CIBERER), U-730, Hospital Duran i Reynals,3ª Planta, Gran Via de L’Hospitalet, 199, E-08907-L’Hospitalet de Llobregat, Barcelona, Spain; 5 Section of Genetics, Phisiological Sciences II Department, Medicine Faculty of Bell vitge, University of Barcelona, Feixa Llarga, sn. 08907 L’Hospitalet de Llobregat, Barcelona, Spain; 6 Department of Medicine, Università degli studi di Padova, Via Giustiniani 2, Padua, Italy; 7 National eScience Centre, 246D, Kelvin Building, Glasgow G12 8QQ, UK; 8 Department of Paediatrics, Medical University of Lodz, 4 Kosciuszki Avenue, Lodz PL-90-419, Poland; 9 Alström Syndrome UK, 49 Southfield Avenue, Paignton, S. Devon TQ3 1LH, UK; 10 IRCAN UMR7284 / INSERM U1081 / UNS, UFR Medecine, Universite Nice Sophia-Antipolis, NICE cedex 2 06107, France; 11 Centrum für Reproduktionsmedizin und Andrologie, WHO Kollaborationszentrum, EAA, Ausbildungszentrum, Universitätsklinikum Münster, Domagkstraße 11, Münster 48149, Germany; 12 The University of Melbourne, Level 3, Doug McDonell Building, Parkville VIC 3010, Australia; 13 Tartu University Children’s Hospital, Lunini 6, Tartu 51014, Estonia; 14 Department of Audiology, H:S Bispebjerg Hospital, Bispebjerg Bakke 23, Copenhagen, NV DK- 2400, Denmark; 15 Wilhelm Johannsen Centre for Functional Genome Research, Department of Cellular and Molecular Medicine (ICMM), The Panum Institute, University of Copenhagen, Blegdamsvej 3, DK-Copenhagen N, Denmark; 16 School of Clinical and Experimental Medicine, College of Medical and Dental Sciences, University of Birmingham, Birmingham B15 2TT, Edgbaston, UK

**Keywords:** Wolfram, Alström, Bardet-Biedl, Diabetes, Patient registries, Rare diseases

## Abstract

**Background:**

Wolfram, Alström and Bardet-Biedl (WABB) syndromes are rare diseases with overlapping features of multiple sensory and metabolic impairments, including diabetes mellitus, which have caused diagnostic confusion. There are as yet no specific treatments available, little or no access to well characterized cohorts of patients, and limited information on the natural history of the diseases. We aim to establish a Europe-wide registry for these diseases to inform patient care and research.

**Methods:**

EURO-WABB is an international multicenter large-scale observational study capturing longitudinal clinical and outcome data for patients with WABB diagnoses. Three hundred participants will be recruited over 3 years from different sites throughout Europe. Comprehensive clinical, genetic and patient experience data will be collated into an anonymized disease registry. Data collection will be web-based, and forms part of the project’s Virtual Research and Information Environment (VRIE). Participants who haven’t undergone genetic diagnostic testing for their condition will be able to do so via the project.

**Conclusions:**

The registry data will be used to increase the understanding of the natural history of WABB diseases, to serve as an evidence base for clinical management, and to aid the identification of opportunities for intervention to stop or delay the progress of the disease. The detailed clinical characterisation will allow inclusion of patients into studies of novel treatment interventions, including targeted interventions in small scale open label studies; and enrolment into multi-national clinical trials. The registry will also support wider access to genetic testing, and encourage international collaborations for patient benefit.

## Correspondence

Wolfram, Alström and Bardet-Biedl (WABB) syndromes are rare, monogenic, autosomal recessive disorders. They are chronically debilitating, highly complex, and in common with other rare diseases, often subject to misdiagnosis, delayed diagnosis, and non-diagnosis. The syndromes exhibit clinical overlap: all can cause blindness, deafness, and diabetes mellitus or impaired glucose tolerance. With 0.57, 0.14 and 0.8 cases per 100,000 [1] respectively, all three WABB syndromes also fall within the EU rare disease definition of ‘a prevalence of not more than 5 affected persons per 10,000 population’ [2].

WABB diseases therefore form part of the estimated 5,000 – 8,000 recognized rare diseases in the EU, spanning a range of clinical specialties, and affecting an estimated 29 million people [3]. Recommendations issued by the European Council in 2009 highlight the need for coordination and cooperation, and networking of resources throughout Europe [4]. A number of projects including Orphanet, EUROPLAN and EURORDIS have made progress in this field. By building on the existing generic frameworks and platforms for rare diseases, Euro-WABB will operate at a disease-specific level to support efficient diagnosis, treatment, and research for WABB diseases in Europe.

### Wolfram syndrome

Wolfram syndrome (OMIM 222300) is characterized by young onset diabetes and bilateral optic atrophy. It is also known as the DIDMOAD, for the other features of the disease including diabetes insipidus, diabetes mellitus, optic atrophy, and deafness. Its prevalence was estimated at 1 in 770,000 in the United Kingdom [5]. Insulin dependent diabetes usually occurs as the initial manifestation during the first decade of life, with onset of the other features in the second and ensuing decades [6]. Neuroradiological examinations show widespread neurodegeneration with severe brainstem atrophy [7,8]. The causative gene, WFS1 encodes Wolframin, a transmembrane protein located in the endoplasmic reticulum (ER) [9-11]. WFS1 serves as an ER calcium channel and has an important function in maintaining homeostasis of the ER in pancreatic β-cells. More than 120 mutations in WFS1 have been identified in patients and most of them are inactivating (nonsense or frameshift) mutations. Fairly recently, it has become evident that the transmission mode of disease-associated *WFS1* mutations may behave either dominantly or recessively and that the onset of medical symptoms may vary tremendously in age of appearance and in degree of severity [12].

### Alström syndrome

Alström syndrome (ALMS) (OMIM 203800) is characterized by early onset blindness and obesity [13]. In addition to the blindness, deafness, obesity and insulin resistance originally described, cardiomyopathy, hyperlipidaemia, renal failure and hepatic fibrosis have been reported [14-19]. The syndrome is caused by loss of function mutations in *ALMS1*, encoding a protein of unknown function located in basal bodies and connected with ciliary function [20-22]. Alstrom patients, in contrast to many patients with Bardet Biedl syndrome, had mostly normal intelligence and absence of polydactyly. The condition is first apparent as photophobia and nystagmus, leading to progressive visual loss from childhood. Obesity, insulin resistance, and partial nerve deafness follow, then type 2 diabetes (80%), severely high blood triglycerides [19], cardiomyopathy (50%), with more than 25% affected in infancy, and renal failure [18]. A smaller number also develop dyssynergy of the bladder resulting in incontinence. Some adult patients develop skeletal abnormalities.

### Bardet-Biedl syndrome

Bardet-Biedl syndrome (BBS) (OMIM 209900) is characterized by early onset retinal dystrophy, obesity, and frequent polydactyly [23]. Associated features can include childhood onset blindness, cystic kidneys, renal failure, global learning difficulties, urological problems, and neurological deficits including sensorineural deafness. Insulin resistance is common and Type 2 diabetes usually develops from puberty onwards. One third of patients will develop renal failure and about 10% will progress to end-stage renal failure requiring dialysis and/or transplantation. There are currently 17 genes associated with BBS [24]. This genetic heterogeneity means that it is not possible to distinguish causative genes based on clinical grounds alone.

### Other syndromes

There remain some rarer syndromes for which diabetes mellitus is a significant part. These include Thiamine responsive megaloblastic anaemia syndrome (TRMA) (also known as Rogers syndrome) and Wolcott-Rallison syndrome. Both are early onset, rare autosomal recessive disorders. TRMA (OMIM 249270) is characterized by Megaloblastic anaemia, diabetes mellitus and sensorineural deafness and Wolcott-Rallison (OMIM 226980) by insulin-dependent diabetes occurring during neonatal and early infancy [25,26]. The registry will capture information about these cases alongside the WABB syndromes.

### EURO-WABB research group

The EURO-WABB research project is managed by a collaboration of European scientists, clinicians and patient group representatives. Project activities are overseen by the Project Management Committee (PMC), with further guidance from the Scientific Advisory Committee (SAC). The project is supported by a number of collaborating partners, spanning a range of different stakeholders, and also benefits from the insight provided by Alström Syndrome UK Patient Support Group and the French Wolfram Association.

The study objectives are as follows:

1. **Establishing the natural history of WABB diseases.**

Registry data will be used to better define disease characteristics and outcomes. Specifically, the study aims to identify whether the natural history varies between population groups and how it changes over time. This information will be used to ascertain whether it is possible to predict from the natural history of these diseases, groups of affected individuals with different outcomes, and also stages during their disease when they may be amenable to intervention.

2. **Assessment of clinical effectiveness of management and quality of care.**

Assessment of clinical effectiveness of management and quality of care. It is likely that there are It is likely that there are disparities in the health care provision and clinical management of WABB syndromes within the EU Member States. Reassessment of existing and potential patients will be used to refine the agreed diagnostic criteria and to facilitate the development of consensus referral, care and management pathways. This will include the identification of requisite service components for optimal disease management along with the associated indicative costs. Given that it is likely that assessment of clinical effectiveness will identify knowledge gaps or specific learning needs of healthcare professionals, educational materials and training tools will be produced and disseminated to target groups.

3. **Development of a European cohort.**

Research into rare diseases such as Wolfram, Alström and Bardet-Biedl is limited by the lack of a patient population of sufficient size from which to develop an evidence-base. Seeking to achieve Europe-wide collaboration, the EURO-WABB registry will provide a large-scale source of observational data to facilitate aetiological research and to support the identification of sub-populations for future interventional research.

4. **Genotype-phenotype correlations.**

The study will investigate whether the genetic mutation can predict the natural history of the disease and, in turn, whether this genetic diagnosis can be used to predict an individual patient outcome. Genotypic differences between WABB patients with an identified mutation and those presenting with a partial form phenotype will also be assessed. To support these activities, the registry will be linked to an open access, comprehensive mutation database stored and catalogued using the Leiden Open source Variation Database (LOVD) web-database software package [27]. The database lists all known pathogenic mutations (published and unpublished) and is updated as new mutations are identified. We also have included on the consent form, the facility to collect tissue samples including blood for RNA extraction. This will aid the interpretation of mutations through assessment of their function at the RNA level.

5. **Increased and Equal Access to Genetic Diagnostic Testing.**

For participants who haven’t undergone genetic testing to confirm diagnosis, and where the cost of genetic testing is not met by national health funding, this will be provided through the project. Access to diagnostic testing will be via clinician referral, ensuring that any resulting diagnosis is coupled with appropriate counseling. Testing is provided through a series of nationally accredited European diagnostic laboratories and follows the pan-European approach outlined in the recommendations by the European Society of Human Genetics Public and Professional Policy Committee (ESHG PPPC) [28,29]. Nine laboratories currently undertaking diagnostic testing for WABB diseases, and who are willing and able to support this aim, have been identified. With their expertise, and supported by project funding, Euro-WABB aims to offer equal access to testing across Europe. As a consequence, this may also lead to the identification of novel mutations, particularly in geographical populations who haven’t previously been able to access testing.

## EURO-WABB project

**Figure 1:**
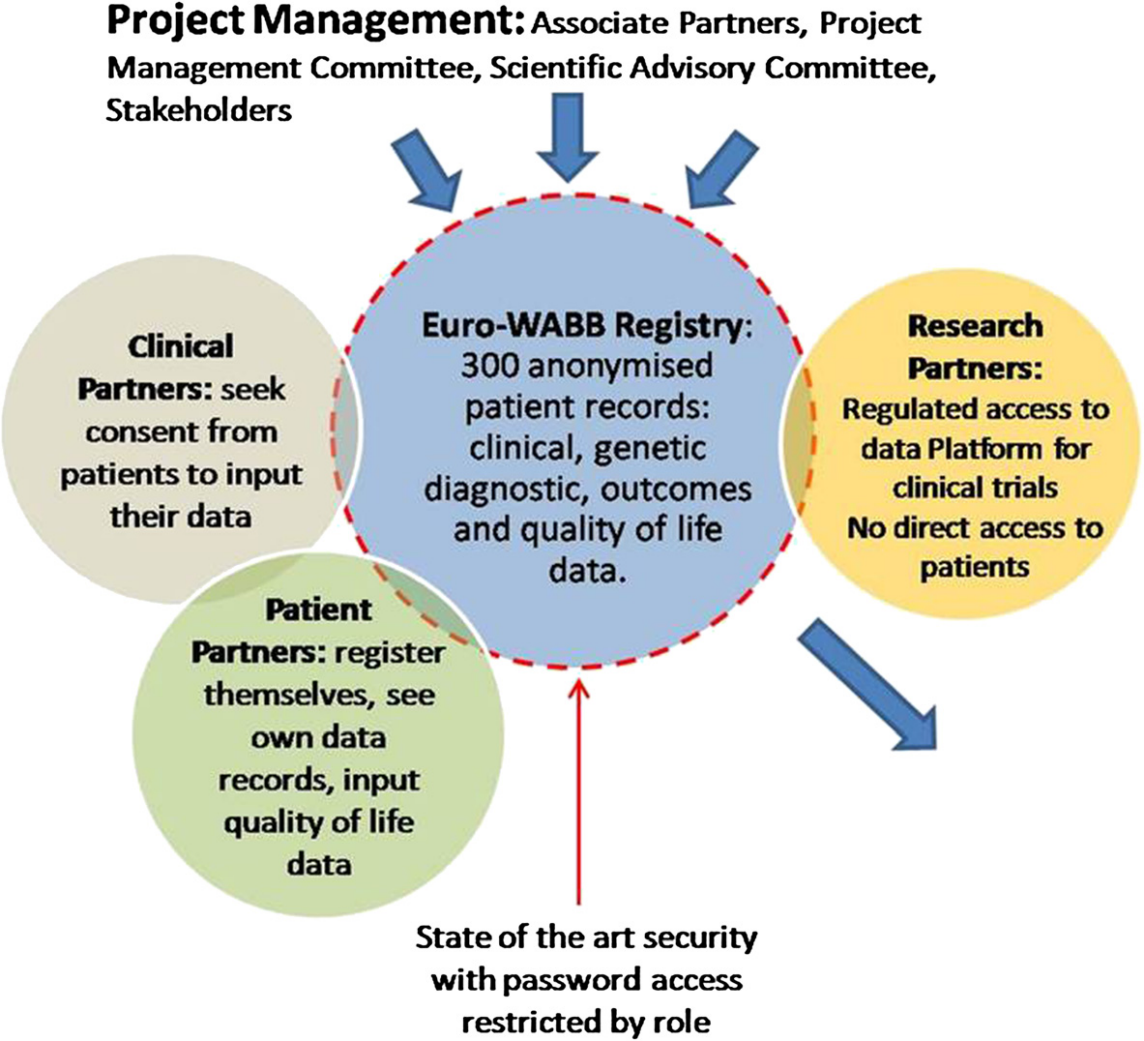
Cartoon of the Euro-WABB registry project.

Euro-WABB is a multi-centre observational study. A comprehensive anonymous registry of children and adults diagnosed with Wolfram, Alström, Bardet-Biedl or other rarer diabetes syndromes from EU Member States will be compiled. Registry data will be split between a ‘Core’ (minimum), ‘Extended’ and ‘Patient Experience’ datasets (Additional file 1). The core dataset includes 44 variables, consisting of summary current clinical and demographic data, and capturing the clinical and genetic data necessary to establish diagnosis. A further 370 variables capturing detailed phenotypic data are included in the extended dataset recording disease progression and date of onset of symptoms form the extended dataset. Patient experience data will record the patient’s journey and their experiences from onset of symptoms, through diagnosis and the subsequent management of their condition (Figure 1)*.*

Clinical diagnostic data across datasets will be standardised using the International Classification of Diseases (ICD) coding system (version 10). The endocrine and hormone sub-set data will be further classified using the European Society for Paediatric Endocrinology (ESPE) Classification of Paediatric Endocrine Diseases.

Data records will be catalogued using a unique identifier, with only the clinician caring for the participant able to link the data record to his/her patient. Electronic data validation systems will ensure that data sets conform to the agreed data models and the quality and adequacy of the submitted data will be regularly reviewed from a clinical perspective. Housed within the overarching VRIE, the database will offer secure access to, and sharing of, data at local, national, and EU level. Access rights and user privileges are determined by role/attribute control based models, with clinical partners and patient partners contributing data to the data record.

### Participants

A minimum of 100 subjects for each WABB disease, plus 50 subjects with a diagnosis of Wolcott-Rallison syndrome or Thiamine Responsive Megaloblastic Anaemia (TRMA), ~350 in total, will be recruited over 36 months. The study sample will include both adults and children, with no upper or lower age limits for data subjects. The clinician caring for the patient will need to confirm eligibility and written informed consent will be obtained prior to registration for all participants. Eligibility is diagnosis driven, with any individual with a clinical diagnosis of WABB or other rarer diabetes syndrome eligible to take part. This eligibility is further widened to include patients who have an identified mutation in a W/A/BB gene(s) but who may not exhibit the described phenotype for the associated syndrome.

### Data collection

**Figure 2:**
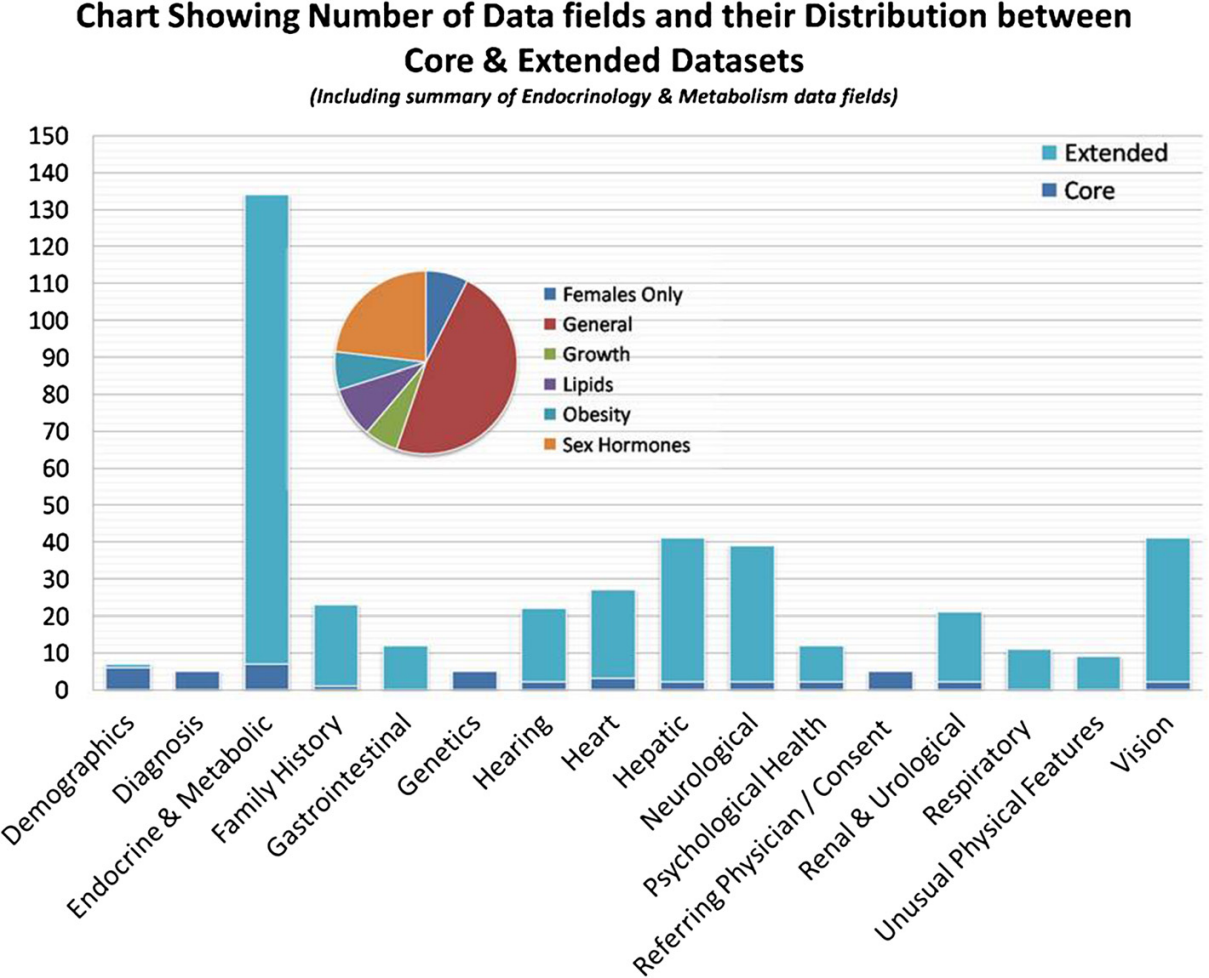
Data fields held in the registry.

The registry database is web-based and clinicians or delegated site staff will enter clinical registry data using a browser-based form. The core and patient experience datasets will be completed once, with the extended dataset completed at repeating intervals generating comprehensive longitudinal data. Affected people or their legally responsible carers will also be able to view and enter data into the registry, provided that their core data is first validated by their clinician (Figure 2).

### Data analysis

The core and extended datasets will include demographics, family history, details of diagnosis, eye signs, deafness, diabetes, associated symptoms, quality of life and investigations. A descriptive analysis of all variables will be carried out first to determine the nature of variable distribution. Each study participant will be categorized where known by core diagnosis (Wolfram, Alström, Bardet-Biedl, other) and then by genotypes (for instance nonsense mutations, missense mutations). Phenotypic information will include the age of onset of diabetes mellitus, optic atrophy, deafness, renal or cardiac complications; and longitudinal data entries on progression of disease as to new onset of symptoms that were not present at baseline data entry. From a biostatistical perspective, the phenotypic information falls into three groups: – continuous, categorical (binary), or the time to event. The data analyses include the unadjusted and adjusted comparisons of the clinical characteristics among the genotype groups. In adjusted analysis, the investigators will quantify the effect of genotype on clinical characteristics after controlling for potential confounders.

For continuous data, we will use the age of onset as an example for description of analyses. A simple ANOVA will be used to compare the means of age onset among the genotypic groups for unadjusted analysis. For binary data (Yes/No), the Chi-square test will be used to compare the proportion of a particular characteristic among the genotype groups.

For the time to event data, a Kaplan Meier time-to-event curve will be generated for each genotype group with the logrank test for statistical significance. The adjusted analysis will be performed using the Cox Proportional Hazard Model.

## Ethical & regulatory considerations

The study was granted ethical approval by The National Research Ethics Service West Midlands Committee – Staffordshire, REC reference 11/WM/0127 in August 2010, and a substantial amendment approved on 22^nd^ January 2013.

### Recruitment of vulnerable groups

The sample population includes a number of vulnerable groups, including adults lacking capacity to give consent, minors and those with special communication needs, e.g. those with a visual or hearing impairment. Detailed participant information will be provided, in a variety of formats, and where appropriate the potential participant’s parent, legal guardian or a consultee will be included in the information giving and consent seeking processes. Additionally, given that WABB syndromes may significantly shorten life expectancy, it may also be appropriate to collect data relating to deceased patients. This will be subject to consent from the deceased person’s next of kin, and only where the clinician feels that this will not be unduly distressing to the deceased person’s family.

## Summary

A common core dataset for European Union states has been developed that can be shared between national rare disease registries as they are established; and will allow linkage with other international disease registries. Agreement on this core dataset for WABB and other very rare diabetes syndromes is essential in order to compare data between national registries, link registries and to identify subgroups of patients that may be eligible for clinical trials or to prioritise genes for mutation searches.

Achieving true Europe-wide collaboration may prove challenging. High usage of the registry will be achieved by linking it to rapid genetic testing; and to up to date, accurate information, FAQS, and education material. Additional awareness raising activities such as dissemination of promotional materials and the development of a multi-language project website (http://www.euro-wabb.org) and dissemination of promotional materials will also be undertaken to help to overcome this challenge.

To encourage maximum participation by health professionals, and in recognition that not all partners will want others to see anonymised clinical data on their own patients, we have included an option to restrict access to their own data. We sincerely hope that most clinical partners will allow their anonymised data to be shared with bona fide researchers working on approved study protocols. Any group of researchers or a pharmaceutical company can apply to the Project Steering Group for access to data in the registry to investigate one or more of these diseases. If permission is granted, then data can be made available on all those patients for whom the clinical partner has granted permission to share data. The data can be restricted to one particular rare disease, to the core datasets, or to system specific data for instance endocrine, cardiac or genetic data.

There are some existing registries for these diseases, based in North America. There are also subspecialty specific databases, that have good penetration with certain groups of health professionals. Euro-WABB has good penetration with endocrinologists, diabetologists and geneticists. There are other registries, such as for deafness, that may also include affected patients with these conditions. In addition, some patient groups set up their own disease registry. Pharmaceutical companies may develop a disease specific registry to determine natural history and monitor outcomes of novel treatments. There may be a case for a specific rare disease to be included in more than one registry, in order to maximize penetration among different interest groups. Given the ultra-rare nature of these diseases, it is important that all anonymised data is shared for maximum patient benefit. To this end we have included on the consent forms, specific consents to allow sharing of data with national, European, and other international disease registries. It is hoped that use of the ICD10 codes, will support shared definitions of symptoms to allow comparison and pooling of data between registries.

It is also recognized that there will need to be more than one entry route to achieve maximum participation in this European registry. Some affected families will research their own condition through internet search engines and find the European registry. We have therefore incorporated a route to allow patients to self register and submit core data and quality of life data about themselves. By asking for contact details for their local doctor, we hope to be able to approach that person to invite to participate as a clinical partner.

We believe in principle that patients should be able to see what data is held on themselves, but not other patients, electronically. We have consulted widely with patient groups and therefore have incorporated a secure system to allow patients to see their own core data online. The extended dataset includes data that is more complex, and may require some explanation by the local physician. We therefore encourage patients who would like to see their own extended dataset, to approach their local physician to view this.

In line with existing evidence and recommendations highlighting that rare disease registries can lead to improved recognition, correct diagnosis, and earlier treatment for patients [30,31], it is hoped that Euro-WABB will lead towards quality of life improvements through earlier diagnosis, prompt identification and management of complications. The registry will develop collaborative links which will be the precursor to a European Reference Network for these diseases.

## Abbreviations

BBS: Bardet-Biedl syndrome; LOVD: Leiden open variation database; PMC: Project management committee; SAC: Scientific advisory committee; TRMA: Thiamine responsive megaloblastic anemia; WABB: Wolfram, Alström and bardet-biedl; VRIE: Virtual research and information environment.

## Competing interests

The authors declare that they have no competing interests.

## Authors’ contributions

All listed authors have contributed significantly to the conception, design and implementation of the study and also in the preparation of the manuscript. All authors have read and approved the final manuscript.

## Pre-publication history

The pre-publication history for this paper can be accessed here:

http://www.biomedcentral.com/1471-2431/13/130/prepub

## Supplementary Material

Additional file 1 List of data fields in core and extended datasets. Click here for file
